# Gender differences in suicide attempters: a retrospective study of precipitating factors for suicide attempts at a critical emergency unit in Japan

**DOI:** 10.1186/1471-244X-14-144

**Published:** 2014-05-19

**Authors:** Ryuichiro Narishige, Yoshitaka Kawashima, Yasushi Otaka, Takuya Saito, Yoshiro Okubo

**Affiliations:** 1 Department of Neuropsychiatry, Nippon Medical School, 1-1-5 Sendagi, Bunkyo-ku, Tokyo 113-8603, Japan; 2 Department of Neuropsychopharmacology, National Institute of Mental Health, National Center of Neurology and Psychiatry, 4-1-1 Ogawahigashi-cho, Kodaira-shi, Tokyo, Japan; 3 Department of Child and Adolescent Psychiatry, Graduate School of Medicine, Hokkaido University, North 15, West 7, Kita-ku, Sapporo-shi, Japan

**Keywords:** Suicide attempters, Precipitating factors for suicides, Gender differences, Critical care medical center

## Abstract

**Background:**

There is a shortage of empirical data concerning precipitating factors for suicides in Japan. The purpose of the present study was to clarify gender differences of precipitating factors for suicide attempts in Japan.

**Methods:**

The subjects were high-lethality suicide attempters who were admitted to the Nippon Medical School Hospital Critical Care Medical Center between March 1, 2010 and March 31, 2012. Precipitating factors for suicide attempt, method of suicide attempt, psychiatric diagnoses and other sociodemographic data were collected from the patients’ medical records retrospectively, and statistical analyses were performed for categorical variables of male/female.

**Results:**

The total number of subjects was 193 (88 males and 105 females). The rate of subjects attempting suicide by poisonous gas was significantly higher in males while that of subjects attempting suicide by drug overdose was significantly higher in females. The rate of subjects diagnosed with “major depressive disorder, bipolar disorder” was significantly higher in males while that of subjects diagnosed with “personality disorders” or “dysthymic disorder” was significantly higher in females. Subjects with “health problems”, “financial problems”, “work problems”, “debts (others)” or “unwanted transfer” were significantly more numerous among males; subjects with “family problems”, “parent–child relations” or “loneliness” were significantly more frequently found among females.

**Conclusions:**

Mental disorders were the most common precipitating factor for suicide attempts regardless of gender. Significant gender differences were observed in psychiatric diagnoses, methods of suicide attempt and psychosocial problems. This indicates the necessity of suicide prevention measures corresponding to these gender differences.

## Background

In Japan, the number of suicides has been decreasing in recent years but has remained at around 30,000 annually after a sudden increase in 1998. Suicide prevention measures have been taken by the whole nation to reduce suicides after the Basic Act on Suicide Prevention was enacted in 2006. On the other hand, it is difficult to narrow down which measures should be taken intensively because empirical data regarding precipitating factors for suicides are insufficient in Japan.

Some previous studies have endeavored to clarify the characteristics of suicide-related behaviors in Japan by investigating suicide attempts [1-4]. However, in those studies, investigations focused only on psychiatric diagnoses or sociodemographic data of suicide attempters, and precipitating factors for suicide attempts were not examined. Pompili argued that suicide is better understood as a phenomenon centered in the individual [5]. Analysis of subjective motives as well as analysis of objective risk factors is useful for understanding suicide. The National Police Agency (NPA) yearly announces the annual number of suicides, and analysis of the precipitating factors for suicides based on the NPA’s suicide statistics is made public simultaneously. However, it is possible that the actual state of suicides has not been grasped correctly in this analysis due to inadequate psychiatric assessment, and precipitating factors for suicides were specified in only about 70% of suicide committers in the NPA’s suicide statistics [6].

Gender differences in suicide-related behavior are well-known. Females have a higher rate of suicide attempts than males, while males have a higher rate of mortality from suicide than females [7-9]. The male/female ratio of suicide attempts is 0.65-0.81 according to previous studies in Japan [1-4]. Females are more likely to attempt suicide by poisoning than males, while males are more likely to use methods of suicide with high lethality like hanging than females [8,9]. In addition to these characteristics, some gender differences in sociodemographic characteristics of suicide committers or attempters are known. Regarding the sociodemographic characteristics of suicide committers in Denmark, Qin et al. reported that being single, unemployed and having a low income were associated with higher suicide risk in males, whereas having a young child was associated with lower suicide risk in females [10]. Fekete et al. reported sociodemographic characteristics of suicide attempters in Hungary, including that females were economically inactive or widowed and males were unemployed or living alone [11]. Zhang et al. studied the sociodemographic characteristics of suicide attempters in American young adults, reporting that low income and smoking were associated with suicide attempts in males, and poor self-evaluated health, low educational attainment and drug use were associated with suicide attempts in females [12]. However, gender differences of precipitating factors for suicide-related behavior have been insufficiently investigated. Wu et al. reported that unemployment or economic problems were regarded as significant precipitating factors for suicide attempts to a greater extent in males than in females in Taiwan, but the sample consisted mainly of people with a suicide lethality ranging from mild to moderate [13]. Tóth et al. reported that interpersonal conflict was found to be the most frequent precipitating factor for suicide attempts by deliberate self-poisoning in Hungary and also that male suicide attempters with interpersonal conflicts had lower levels of depression [14].

In the present study, we investigated precipitating factors for suicide attempts from the data of suicide attempters admitted to the Nippon Medical School Hospital Critical Care Medical Center. Gender differences in Japanese suicide-related behavior have not been clarified. Thus, we aimed to clarify the gender differences in precipitating factors for suicide attempts in Japan.

We previously reported a study concerning precipitating factors for suicide attempts based on a preliminary psychiatric assessment of suicide attempters, in which we explored the precipitating factors for suicide attempts among adolescent suicide attempters [15]. In the present study, we comprehensively investigated precipitating factors for suicide attempts via an approach unprecedented in Japan.

## Methods

### Study design and sample

The subjects were suicide attempters with high lethality who were admitted to the Nippon Medical School Hospital Critical Care Medical Center. All patients admitted to this facility are in medically serious and fatal condition. About 2,000 patients are admitted every year, and about 5% of them are suicide attempters excluding suicide committers. The study period was from March 1, 2010 to March 31, 2012.

In this report, the term “suicide attempt” follows the Columbia Classification Algorithm of Suicide Assessment (C-CASA), i.e., it is defined as a potentially self-injurious behavior, associated with at least some intent to die, as a result of the act [16]. We adopted this definition of the term “suicide attempt” because it is often clinically used. Silverman et al. proposed the nomenclature for suicidology, defining “suicide attempt” as a self-inflicted, potentially injurious behavior with a nonfatal outcome for which there is evidence of intent to die [17,18]. There is no essential difference between C-CASA and the nomenclature proposed by Silverman et al. in respect to the definition of the term “suicide attempt”.

At the Nippon Medical School Hospital Critical Care Medical Center, psychiatrists examine all suicide attempters and confirm their intent to die when they attempted suicide. Psychiatrists assess their mental state and problems that precipitated their suicide attempt. Psychiatric diagnoses were made according to the DSM-IV-TR criteria [19] by agreement among two or more experienced psychiatrists. Psychiatrists also assessed precipitating factors for the suicide attempts. Precipitating factors for suicide attempt, methods of suicide attempt, psychiatric diagnoses and other sociodemographic data were collected from the patients’ medical records retrospectively. If the subject had attempted suicide by two or more methods, we only presented the most lethal one.

Precipitating factors for suicide attempts were classified in accordance with the items used for the classification of precipitating factors for suicides by the NPA’s 2010 suicide statistics (Table 1) [6]. These are the only official statistics that include data on precipitating factors for suicides, and they are widely used as the basic data for suicide prevention in Japan. This is why the classification by the NPA’s suicide statistics was used in the present study. Three items or less were presented as precipitating factors for suicides in the NPA’s 2010 suicide statistics, while more than three items were also presented as precipitating factors of suicide attempts in the present study in order to show the background of suicide attempts more accurately. The items for mental disorders in the NPA’s 2010 suicide statistics (“depression”, “schizophrenia”, “alcohol dependence”, “drug abuse” and “other mental disorders”) were removed from the items in the present study because psychiatric diagnoses were made according to the DSM-IV-TR criteria independently.

**Table 1:** Items used for classification in NPA’s suicide statistics, 2010

**Major category**	**Sub-classification**
Family problems	Parent–child relations, marital relations, relations with other family members, death in the family, pessimism over family’s future, scolding from family members, worries about parenting, abuse, exhaustion from nursing or care, others
Health problems	Disease, depression, schizophrenia, alcohol dependence, drug abuse, other mental disorders, physical handicaps, others
Financial problems	Bankruptcy, business slump, redundancy, failure to find employment, poverty, debts (accumulated loans), debts (joint liability), debts (others), repayment pressure from creditors, insurance paid by suicide, others
Work problems	Failure in business, workplace relationship, unwanted transfer, work overload, others
Love problems	Marriage problems, broken heart, worries about adultery, trouble with boyfriend or girlfriend, others
School problems	Entrance examinations, career decisions, academic failure, relationship with teachers, bullying, relationship with schoolmates, others
Other problems	Having ones’ crime revealed, crime victim, suicide over someone’s death, loneliness, relationship with neighbors, others

If there were two or more precipitating factors belonging to the same major category in one subject, the number of precipitating factors in that major category was considered to be one only when the analysis was performed between major categories.

### Statistical analyses

Statistical analyses were conducted using PASW Statistics 18 (SPSS Inc., Chicago, IL, USA). Welch’s t-test was used to compare the variables of age and the number of precipitating factors between males and females. Chi-square test or Fisher’s exact test was used to determine the categorical variables of being under psychiatric treatment, employment status, method of suicide attempt, psychiatric diagnosis and precipitating factors for suicide attempt between males and females. Chi-square test or Fisher’s exact test was also used to determine the categorical variables of method of suicide attempt between under psychiatric treatment and no psychiatric treatment. We used a significance level of p < 0.05 and two-sided probability.

### Ethics

The present study was approved by the Ethics Committee of Nippon Medical School Hospital and conforms to the provisions of the Declaration of Helsinki.

## Results

### Characteristics of subjects

The results are shown in Table 2.

**Table 2:** Precipitating factors of suicide attempts and gender differences

	**Total**	**Males**	**Females**	**Significance**	
	**(N = 193)**	**(N = 88)**	**(N = 105)**		
Age ± SD	41.1 ± 16.3	42.4 ± 16.3	40.1 ± 16.4	NS^§^	
Mean number of precipitating factors ± SD	1.11 ± 0.78	1.20 ± 0.87	1.04 ± 0.69	NS^§^	
Under psychiatric treatment	131	50	81	*p* = 0.003^¶^	χ^2^ = 9.070
**Employment status**					
Employed	60	39	21	*p* = 0.000^¶^	χ^2^ = 13.216
Unemployed	97	39	58	NS^¶^	
Housewife or house-husband	17	0	17	*p* = 0.000^¶^	χ^2^ = 15.624
Student	19	10	9	NS^¶^	
**Methods of suicide attempt**					
Drug overdose	101	36	65	*p* = 0.004^¶^	χ^2^ = 8.460
Jumping from a high place	33	17	16	NS^¶^	
Cutting	16	10	6	NS^¶^	
Poisonous gas	15	13	2	*p* = 0.001^¶^	χ^2^ = 11.060
Hanging	11	7	4	NS^¶^	
Poisoning	9	4	5	NS^†^	
Other methods	11	2	9	NS^¶^	
**DSM-IV-TR**					
Substance-induced disorders	16	11	5	NS^¶^	
Schizophrenia and other psychotic disorders	45	22	23	NS^¶^	
Major depressive disorder, bipolar disorder	42	26	16	*p* = 0.016^¶^	χ^2^ = 5.756
Dysthymic disorder	26	7	19	*p* = 0.040^¶^	χ^2^ = 4.224
Adjustment disorders	27	13	14	NS^¶^	
Personality disorders	22	2	20	*p* = 0.000^¶^	χ^2^ = 13.339
Other psychiatric disorders	14	6	8	NS^¶^	
None	11	6	5	NS^¶^	
**Precipitating factors**					
Family problems	62	20	42	*p* = 0.010^¶^	χ^2^ = 6.551
Parent–child relations	14	2	12	*p* = 0.015^¶^	χ^2^ = 5.965
Health problems	8	7	1	*p* = 0.024^†^	
Financial problems	40	24	16	*p* = 0.040^¶^	χ^2^ = 4.220
Debt (others)	4	4	0	*p* = 0.042^†^	
Work problems	27	18	9	*p* = 0.018^¶^	χ^2^ = 5.618
Unwanted transfer	4	4	0	*p* = 0.042^†^	
Love problems	20	7	13	NS^¶^	
School problems	6	3	3	NS^†^	
Other problems	29	12	17	NS^¶^	
Loneliness	12	2	10	*p* = 0.038^¶^	χ^2^ = 4.317

^§^Welch’s t-test, ^¶^chi-square test, ^†^Fisher’s exact test.

The total number of subjects was 193 (88 males and 105 females), with the male/female ratio being 0.84. Their mean age was 41.1 ± 16.3 SD years (range: 15–91 years). Sixty of the subjects (31.1%) were employed.

Those attempting suicide by drug overdose comprised the largest group, with 101 cases (52.3%). The second largest group, 33 cases (17.1%), consisted of those attempting suicide by jumping from a high place. Hanging is the most common method among suicide committers in Japan, but the subjects who attempted suicide by hanging were the fifth most, with 11 cases (5.7%).

182 of the 193 subjects (94.3%) had mental disorders and 131 of the 193 subjects (67.9%) were under psychiatric treatment. Those diagnosed with “mood disorders” were the most, with 68 cases (35.2%): 36 of them were diagnosed with “major depressive disorder”, 6 with “bipolar disorder”, and 26 with “dysthymic disorder”. The subjects diagnosed with “schizophrenia and other psychotic disorders” were the second largest group, with 45 cases (23.3%). The third largest group, 27 cases (14.0%), consisted of those diagnosed with “adjustment disorders”. The fourth largest group consisted of “personality disorders”, with 22 cases (11.4%): 18 of them were diagnosed with “borderline personality disorder” and 4 with “personality disorder not otherwise specified”.

The mean number of precipitating factors, except mental disorders, was 1.11 ± 0.78 SD per subject (range: 0–4). The subjects with “family problems” made up the largest group, with 62 cases (32.1%). Those with “financial problems” comprised the second largest, with 40 cases (20.7%), and those with “other problems” the third largest, with 29 cases (15.0%).

### Gender differences

There was no significant difference in mean age and mean number of precipitating factors between males and females. The proportion of employed subjects was significantly larger among males (chi-square test, χ^2^ = 13.216, *p* = 0.000). 16.2% of the female subjects were housewives, and there were no house-husbands among the male subjects; the rate of subjects who were housewives or house-husbands was significantly higher in females (chi-square test, χ^2^ = 15.624, *p* = 0.000).

The rate of subjects attempting suicide by drug overdose was significantly higher in females (chi-square test, χ^2^ = 8.460, *p* = 0.004), and the rate of those attempting suicide by poisonous gas was significantly higher in males (chi-square test, χ^2^ = 11.060, *p* = 0.001).

The number of subjects under psychiatric treatment was 50 in males (56.8%) and 81 in females (77.1%), with the rate being significantly higher in females (chi-square test, χ^2^ = 9.070, *p* = 0.003). Table 3 shows the differences in the methods of suicide attempt due to the presence or absence of psychiatric treatment. The rate of subjects attempting suicide by drug overdose was significantly higher in the subjects under psychiatric treatment (chi-square test, χ^2^ = 12.479, *p* = 0.000). On the other hand, the rates of those attempting suicide by cutting (chi-square test, χ^2^ = 4.657, *p* = 0.031) and poisoning (Fisher’s exact test, *p* = 0.032) were significantly lower in the subjects under psychiatric treatment.

**Table 3:** Differences in methods of suicide attempt due to the presence or absence of psychiatric treatment

	**Total**	**Under psychiatric treatment**	**No psychiatric treatment**	**Significance**	
	**(N = 184)**				
		**(N = 131)**	**(N = 62)**		
**Methods of suicide attempt**					
Drug overdose	101	80*	21	*p* = 0.000^¶^	χ^2^ = 12.479
Jumping from a high place	33	24	9	NS^¶^	
Cutting	16	7	9	*p* = 0.031^¶^	χ^2^ = 4.657
Poisonous gas	15	7	8	NS^†^	
Hanging	11	5	6	NS^†^	
Poisoning	9	3	6	*p* = 0.032^†^	
Other methods	11	6	5	NS^†^	

^¶^chi-square test, ^†^Fisher’s exact test.

^*^53 females (81.5%) and 27 males (75.0%) under psychiatric treatment attempted suicide by drug overdose.

The rate of subjects diagnosed with “major depressive disorder” or “bipolar disorder” (chi-square test, χ^2^ = 5.756, *p* = 0.016) was significantly higher in males, and the rate of those diagnosed with “personality disorders” (chi-square test, χ^2^ = 13.339, *p* = 0.000) or “dysthymic disorder” (chi-square test, χ^2^ = 4.224, *p* = 0.040) was significantly higher in females.

Among major categories of precipitating factors, the rates of subjects with “health problems” (Fisher’s exact test, *p* = 0.024), “financial problems” (chi-square test, χ^2^ = 4.220, *p* = 0.040) and “work problems” (chi-square test, χ^2^ = 5.618, *p* = 0.018) were significantly higher in males, whereas the rate of those with “family problems” (chi-square test, χ^2^ = 6.551, *p* = 0.010) was significantly higher in females. Among sub-classifications of precipitating factors, the rate of subjects who had “debts (others)” (“financial problems”) (Fisher’s exact test, *p* = 0.042) or “unwanted transfer” (“work problems”) (Fisher’s exact test, *p* = 0.042) was significantly larger in males; the rate of subjects with “parent–child relations” (“family problems”) (chi-square test, χ^2^ = 5.965, *p* = 0.015) or “loneliness” (“other problems”) (chi-square test, χ^2^ = 4.317, *p* = 0.038) was significantly higher in females.

## Discussion

Previous studies have shown that mental disorders are the most common precipitating factor for suicide-related behavior regardless of gender [3,20,21]. Yamada et al. reported that 95% and 65% of suicide attempters had mental disorders and were under psychiatric treatment, respectively [3]. In the present study, 94.3% of the subjects had mental disorders and 67.9% were under psychiatric treatment, percentages similar to those of the previous study. Pompili et al. reported that suicide in eating disorders is a major cause of death [22], but there was no subject with eating disorders in the present study.

The rate of subjects diagnosed with “major depressive disorder, bipolar disorder” was significantly lower, and that of subjects diagnosed with “personality disorders” and “dysthymic disorder” was significantly higher in females. Isometsä et al. compared suicide committers with unipolar depression not fulfilling the criteria of major depressive disorder with those with major depressive disorder [23]. They reported that problems in recent life events were observed more commonly among suicide committers with non-major depressive disorders, and particularly during the final week. Further, patients with personality disorder, especially borderline personality disorder, typically have high impulsivity. Considering these findings, it is possible that females might tend to attempt suicide without major depressive disorder but rather in connection with life events or impulsivity.

In the present study, the rate of subjects attempting suicide by drug overdose was significantly higher in females. It is known that females are more likely to attempt suicide by poisoning than males [8,9], but we can infer from the high rate of female subjects under psychiatric treatment that they could get drugs for suicide attempts more easily than acquire items for other suicidal methods.

Male suicide attempters tend to be influenced by societal problems like “financial problems” or “work problems” and female suicide attempters by social problems like “parent–child relations” or “loneliness.” These findings indicate that male suicide attempters tended to attempt suicide in societal situations, while female suicide attempters tended to attempt suicide in social situations. This difference between males and females may reflect the structure of Japanese society, in which social participation of females is still insufficient — the recent labor force participation rate of those aged 15–64 years is about 80% in males and about 60% in females [24]. In the present study, the rate of those who were employed was significantly higher in males and the rate of the subjects who were housewives or house-husbands was significantly higher in females.

In the present study, the distinct gender differences were confirmed in psychiatric diagnoses, methods of suicide attempt and psychosocial problems, indicating the necessity of suicide prevention measures corresponding to these gender differences, e.g., support for solving societal problems for males and preventing psychosocial isolation for females.

## Limitations

The main limitation of the present study is that the subjects were suicide attempters, i.e., they did not commit suicide. It could be argued, therefore, that the results may not accurately reflect the characteristics of suicide committers. However, suicide attempt, and especially repetitious suicide attempts, is known as a high-risk factor for subsequent suicide [25-27]. Furthermore, all of the subjects were high-lethality suicide attempters and clearly intended to kill themselves (most suicide attempters admitted to the critical care medical center use highly lethal methods in their suicide attempts), so we believe that they had very similar characteristics to those of persons who committed suicide, indicating that our results truly reflect the characteristics of suicide committers in Japan. In addition, the advantage of investigating suicide attempters is that we can directly confirm precipitating factors for suicide attempts from the attempters themselves, as well as perform psychiatric assessment regarding their mental state at the time of their suicide attempts. Therefore, investigating suicide attempters such as in the present study is considered to be an effective method for clarifying the characteristics of suicide-related behaviors.

Another limitation of the present study is that we collected the subjects’ information from their medical records and did not use objective methods like structured interviews when we assessed their psychiatric diagnoses and precipitating factors for suicide attempts. Instead, we assessed the subjects’ psychiatric diagnoses and precipitating factors for suicide attempts by agreement among two or more psychiatrists.

Previous suicide attempt is known as a risk factor for suicide, while it is not considered to be a precipitating factor for suicide and is not included in the classification items of the NPA’s suicide statistics. Therefore, we did not include previous suicide attempt in the analysis of the present study. This might be a limitation of the present study.

## Conclusions

Mental disorders were the most common precipitating factor for suicide attempts regardless of gender. This indicates the necessity for reinforcement of the mental health system as a basic suicide prevention measure. Gender differences were significantly observed in psychiatric diagnoses, methods of suicide attempt and psychosocial problems. Suicide prevention measures based on these gender differences should be performed.

## Abbreviations

NPA: National police agency; C-CASA: Columbia classification algorithm of suicide assessment.

## Competing interests

The authors declare that they have no competing interests.

## Authors’ contributions

All authors contributed to the conception and design of the study. RN, YK and YO contributed to data collection. RN performed the statistical analyses and wrote the first and final drafts based on a review and comments from all authors listed. All authors read and approved the final manuscript.

## Pre-publication history

The pre-publication history for this paper can be accessed here:

http://www.biomedcentral.com/1471-244X/14/144/prepub
